# Green Synthesis of Antimicrobial Silver Nanoparticles (AgNPs) from the Mucus of the Garden Snail *Cornu aspersum*

**DOI:** 10.3390/molecules30102150

**Published:** 2025-05-13

**Authors:** Maria Todorova, Ventsislava Petrova, Bogdan Ranguelov, Georgy Avdeev, Lyudmila Velkova, Stela Atanasova-Vladimirova, Emiliya Pisareva, Chavdar Tankov, Anna Tomova, Aleksandar Dolashki, Pavlina Dolashka

**Affiliations:** 1 Institute of Organic Chemistry with Centre for Phytochemistry—Bulgarian Academy of Sciences, 1113 Sofia, Bulgaria; krasimirova_m@yahoo.com (M.T.); lyudmila.velkova@orgchm.bas.bg (L.V.); 2 Faculty of Biology, Sofia University “St. Kliment Ohridski”, 1164 Sofia, Bulgaria; vpetrova@biofac.uni-sofia.bg (V.P.); episareva@uni-sofia.bg (E.P.); tankov.chavdar@gmail.com (C.T.); aatomova@biofac.uni-sofia.bg (A.T.); 3 Institute of Physical Chemistry “Rostislav Kaishev”—Bulgarian Academy of Sciences, 1113 Sofia, Bulgaria; rangelov@ipc.bas.bg (B.R.); g_avdeev@ipc.bas.bg (G.A.); statanasova@ipc.bas.bg (S.A.-V.); 4 Center of Competence “Clean Technologies for Sustainable Environment—Water, Waste, Energy for Circular Economy”, 1000 Sofia, Bulgaria

**Keywords:** antimicrobial activity, snail *Cornu aspersum*, ascorbic acid, silver nanoparticles

## Abstract

The green synthesis of metal nanoparticles, mediated by extracts from various biological sources, leads to the formation of nanoparticles with unique characteristics and potential biomedical applications. In this study, a fraction of the mucus from the snail *Cornu aspersum* with MW > 20 kDa was used as a bioreducing and biostabilizing agent to obtain silver nanoparticles (AgNPs) in different pH media (pH 1.5, 3.5, and 7.0). The AgNPs were characterized using UV-visible spectroscopy, SEM, TEM, XRD, and FTIR. The synthesis at pH 1.5 and pH 3.5 in the presence of two reducing agents (i.e., the *C. aspersum* mucus fraction with MW > 20 kDa and ascorbic acid [AsA]) resulted in the formation of well-formed spherical nanoparticles (NPs) with larger sizes (20–80 nm) than the NPs obtained at pH 7.0 (20–60 nm) in the presence of only one reducing agent. Furthermore, the biosynthesized AgNPs significantly inhibited the growth of medically significant pathogens such as Gram-positive (*Bacillus subtilis* NBIMCC2353, *Bacillus spizizenii* ATCC 6633, *Staphylococcus aureus* ATCC 6538, and *Listeria innocua* NBIMCC8755) and Gram-negative (*Escherichia coli* ATCC8739, *Salmonella enteitidis* NBIMCC8691, *Salmonella typhimurium* ATCC 14028, *Stenotrophomonas maltophilia* ATCC 17666) bacteria compared to output mucus.

## 1. Introduction

Metal nanoparticles (MNPs) are of great importance and are attracting increasing interest and application in various areas of life, leading to the development of new, environmentally friendly methods of preparation [1,2,3,4,5,6]. Due to a number of disadvantages, such as the use of harsh chemical conditions and organic solvents, and the production of toxic by-products during their synthesis, traditional physical and chemical methods for the preparation of MNPs are being replaced by an alternative method. This includes the “green synthesis” of nanoparticles (NPs), which uses biological systems to reduce metal ions and stabilize of the resulting MNPs [3,4]. The metals used in green synthesis are copper (Cu), zinc (ZnO), gold (Au), silver (Ag), titanium (TiO_2_), and cobalt (Co) [5]. Silver nanoparticles (AgNPs) have attracted considerable interest due to their physicochemical properties and remarkable applications in catalysis, biomedicine, electronics, etc. [7,8]; however, the toxicity of AgNPs is a major problem for their use in the field of biomedicine [8,9,10].

A number of methods for the synthesis of AgNPs have been published, with the reducing agents being useful microorganisms (bacteria, fungi, or yeasts) that produce enzymes capable of reducing metal ions to nanoparticles [11,12,13]. Thus, secondary metabolites from the actinomycete strains *Microbacterium proteolyticum* LA2 [R] and *Streptomyces rochei* LA2 [O] have been used as reducing agents for the synthesis of AgNPs over 7 days at 37 °C, which proved effective against bacterial pathogens causing meningitis, such as *Neisseria meningitides*, as well as *Streptococcus pneumoniae*, and *Haemophilus influenzae* [11]. Also, AgNPs synthesized from the extracellular components of *Streptomyces hygroscopicus* have proved highly effective against medically significant pathogenic Gram-positive (Gram^+^) bacteria (*Bacillus subtilis* and *Enterococcus faecalis*), Gram-negative (Gram^-^) bacteria (*Escherichia coli* and *Salmonella typhimurium*), and yeast (*Candida albicans*) [12].

Mushrooms have also been found to contain a number of active agents that can reduce silver ions. Sharma et al. described secondary metabolites (terpenoids and phenols) from an extracellular extract of the endophytic fungus *Talaromyces purpureogenus*, which are involved in the synthesis of AgNPs [13].

Plant extracts from leaves, flowers, roots, and fruits, which contain reducing and stabilizing agents (flavonoids, terpenes, alkaloids, etc.) have also been used as reducing systems for silver ions [14,15,16]. The AgNPs synthesized with reducing active components from white radish *Raphanus sativus* var. aegyptiacus are effective against various plant pathogenic fungi and the land snail *Eobania vermiculata*, which is a pest of crops [14]. A simple method for the bioreduction of silver ions from *Ocimum gratissimum* leaf extract and the synthesis of AgNPs, which inhibit biofilm formation by *Escherichia coli* and *Staphylococcus* aureus, has been developed [15]. Also, AgNPs synthesized with an ethanol extract of *Alstonia congensis* leaves have shown molluscicidal activity against the parasites *Physa acuta* and *Bulinus forskalii* [16]. Furthermore, AgNPs synthesized from an aqueous extract of *Acanthospermum australe* and silver nitrate are useful in the treatment of skin infections, suggesting that the combination of traditional medicine and nanotechnology could open the door to innovative strategies [17].

In recent years, combined methods have been increasingly used in green synthesis to obtain AgNPs [18]. AgNPs synthesized by two reducing agents from *Alternanthera sessilis* leaf extract and *Oregano root* extract showed significant inhibitory effects against the clinically important pathogens *B. subtilis*, *S. aureus*, *P. aeruginosa*, and *E. coli* [18]. Also, combined reducing agents, such as *Curcuma longa* leaf extract and *Achatina fulica* shell waste, have been used in the green synthesis of AgNPs, resulting in a nanocomposite effective against *E. coli*, *S. aureus*, *Kliebsiella pneumonia*, and *Streptococcus pyogenes* [6,19].

The microorganism- and plant-mediated biogenic synthesis of metal nanoparticles is gaining immense popularity as an advantage of their use is the presence of secondary metabolites, amino acids, and proteins, which are involved in the steps of nanoparticle synthesis [20,21]. In recent years, the synthesis of MNPs using reducing agents from snails has been presented as a simple, clean, and easily scalable method. Mane et al. presented the ecological synthesis of AgNPs with the mucus from the snail *Achatina fulica* as the reducer [22]. The AgNPs in the mucus matrix exhibited a broad inhibitory spectrum against bacteria, such as *E. coli*, *Staphylococcus aureus*, *Klebsiella pneumoniae*, and *Pseudomonas aeruginosa*, and the fungal strain *Aspergillus fumigatus*, as well as anticancer activity, showing a 15% inhibition in HeLa cells [5,22]. The purified snail mucus used to form biogenic silver nanoparticles contains mainly proteins and glycoproteins, which are very good reducing agents at neutral pH and room temperature [22,23,24,25,26,27,28,29]. Also, Surowka et al. proved that the main reducing agents in the mucus of the small, brown snail *Cornu aspersum aspersum* are located in the foot and visceral sac [23]. It has been suggested that the tyrosine residues of proteins are involved in the mechanism of green synthesis, causing the reduction of silver ions, while macromolecules located around the nanoparticles have a stabilizing role, protecting them from aggregation over time [24].

The mucus of the garden snail *Cornu aspersum*, grown in ecological farms in Bulgaria, has been thoroughly studied, and important components such as glycoproteins, peptides, and metabolites with proven antibacterial and antitumor properties have been identified [25,26,27,28]. In our previous studies, we demonstrated the involvement of these mucus proteins as reducing agents in forming copper nanoparticles (CuNPs) [27,29]. The inclusion of a second reducing agent, ascorbic acid, leads to the green synthesis of copper CuONPs with more pronounced antibacterial and antifungal activity [30].

Based on previous studies on the mucus of the garden snail *C. aspersum*, this article presents the green synthesis of AgNPs-Muc with a reducing and stabilizing agent fraction from the mucus of the snail *C. aspersum*, with bioactive compounds with MW > 20 kDa and proven antibacterial activity. The influence of different preparation conditions, such as the silver nitrate concentration and pH of the medium, was investigated. The AgNPs were characterized using a variety of microscopic and spectroscopic techniques, and their antibacterial activity was also demonstrated.

## 2. Results

### 2.1. Green Synthesis of Silver Nanoparticles from the Mucus Extract of C. aspersa (AgNPs-Muc)

#### 2.1.1. Isolation and Characterization of the Mucus Extract

**Figure 1:**
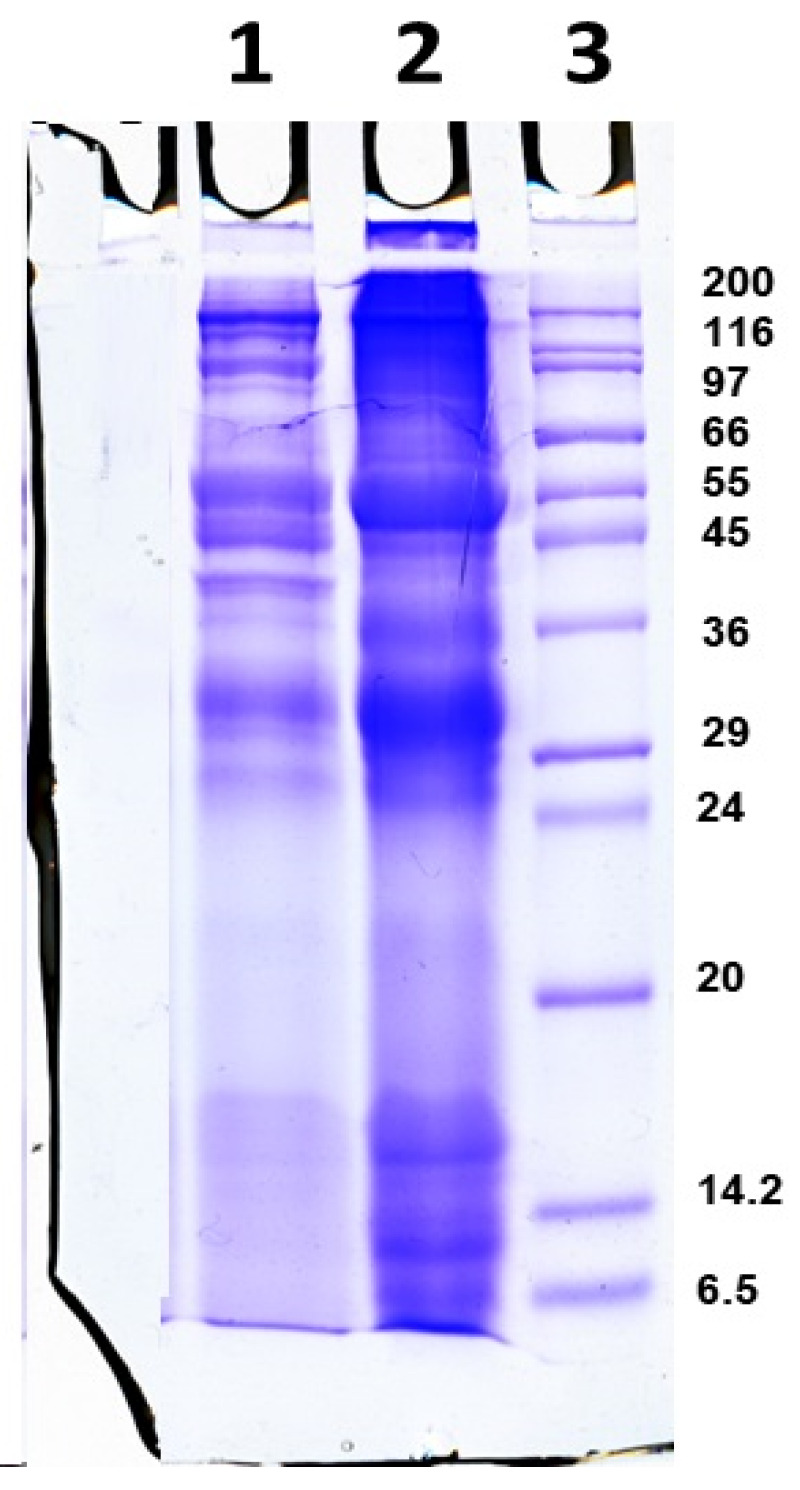
Twelve percent SDS-PAGE of (1) snail mucus with MW > 20 kDa after dialysis of the starting mucus and concentration on 20 kDa membrane, (2) starting mucus before dialysis, and (3) molecular markers of standard proteins with molecular weights ranging from 6.5 to 200 kDa (SigmaMarker^TM^, Sigma-Aldrich, Saint Louis, MO, USA).

The native extract was collected from the mucus of *C. aspersum* snails raised in Bulgarian ecological farms using patented technology [31,32]. After 24 h of dialysis and concentration on a 20 kDa membrane, a purified solution of snail mucus was obtained, which was enriched with proteins with MW above 20 kDa. The purity of the sample and its protein profile were determined using electrophoretic analysis using 12% SDS-PAGE (Figure 1) before (lane 2) and after dialysis and concentration on a 20 kDa membrane (lane 1) as well as the UV–Vis spectra which correspond to those presented by Dolashka et al. [29].

The analysis showed that the mucus contained proteins with molecular weights of 26, 30, 35, 37, 45, 56, 80, and >100 kDa. The total protein concentration in the sample was determined based on the obtained UV–Vis spectrum by measuring the absorbance at 280 nm; however, this method is not the most accurate since it is based on the presence of the amino acids tryptophan, tyrosine, and phenylalanine in the protein composition. After applying the more sensitive Bradford method [33], a total protein concentration of 1.80 mg/mL was determined in the mucus.

#### 2.1.2. Green Synthesis of AgNPs in the Mucus Matrix with MW > 20 kDa

Biogenic AgNPs-Muc were obtained via green synthesis with a reducing fraction with MW > 20 kDa from the mucus of *C. aspersum* and a precursor of Ag^+^ ions (AgNO_3_). The process was monitored three times under different conditions, varying the ratio between the reagents (mucus fraction and AgNO_3_), with one or two reducing agents (mucus fraction and ascorbic acid), and at three different pH conditions (pH 1.5, 3.5, and 7.0). AgNPs were obtained using three main methods, with the common steps involving mixing the mucus fraction with MW > 20 at a concentration of 0.05 mg/mL with 50 mM AgNO_3_ in a ratio of 1:1 with continuous stirring in the dark.

In the first method, a pH of 1.5 was achieved after titration with 250 mM AsA. In the second method, the reaction mixture was titrated with 250 mM AsA until the solution pH reached 3.5. In the third method, the synthesis was carried out at pH 7.0 and with only one reducing agent—the *C. aspersum* mucus fraction. The formation of biogenic nanoparticles was monitored using UV–Vis spectrometric analyses for 3 days with continuous stirring on a magnetic stirrer. To stabilize the AgNPs, after mixing the reagents, the reaction mixture was exposed to sunlight for 30 min in all three methods. After the third day, the biosynthesized AgNPs-Muc were centrifuged at 3000 rpm to remove coarse particles. The resulting precipitates, after washing three times with dH_2_O and additional centrifugation at 12,000 rpm, were dried at 25 °C. The obtained AgNPs-Muc were analyzed using various methods and techniques to determine their structure and properties.

### 2.2. Characterization of the Obtained AgNPs-Muc

#### 2.2.1. UV–Vis Analyses of AgNPs-Muc

**Figure 2:**
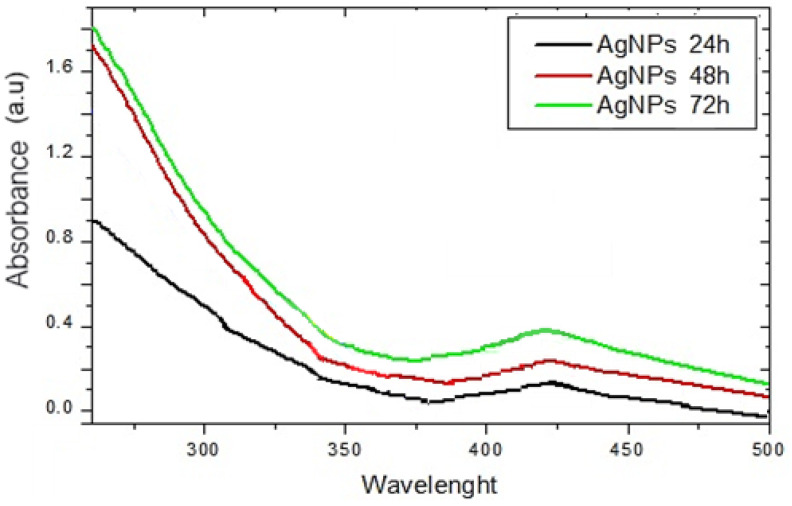
UV–Vis spectra of the synthesized AgNPs-Muc at pH 1.5 after 24 h, 48 h, and 72 h.

The process of reducing the Ag^+^ ions was observed visually during all three methods (pH 1.5, 3.5, and 7.0) by changing the initial color of the reaction mixture from colorless to deep brown. The time-dependent synthesis of AgNPs-Muc was also monitored using UV–Vis spectra recorded after synthesis for 24 h, 48 h, and 72 h. The best-formed nanoparticles were observed after 72 h of synthesis at pH 1.5 (Figure 2). The trend shown in Figure 2 was observed in all three of the formation methods.

#### 2.2.2. Characterization of the AgNPs-Muc Using Scanning Electron Microscopy Combined with Energy Dispersive Spectroscopy (SEM/EDS)

**Figure 3:**
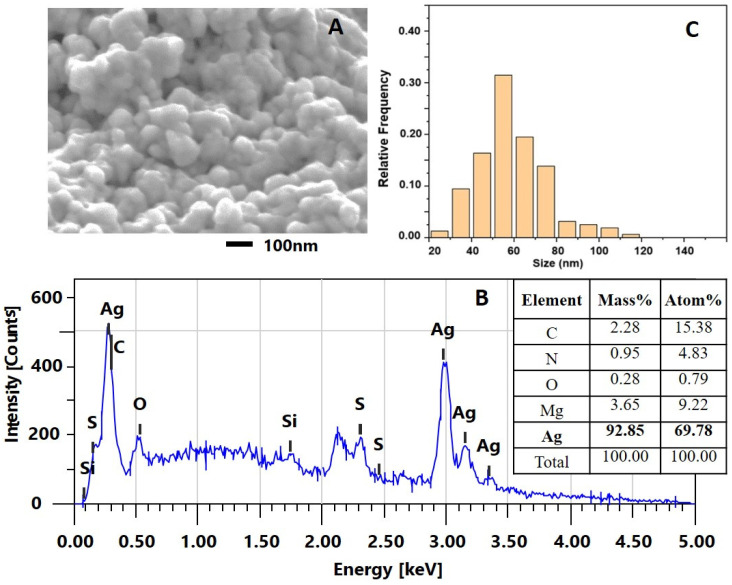
Characterization of AgNPs-Muc obtained by method 1 at pH 1.5. (**A**) Scanning electron microscope image of AgNPs (SEM); (**B**) Energy dispersive X-ray (EDS) spectrum of AgNPs-Muc and summary results table; (**C**) Histogram of the particle-size distribution.

The formed biogenic AgNPs-Muc were imaged using scanning electron microscopy in combination with energy dispersive spectroscopy, and the morphology of the AgNPs-Muc obtained under the different reaction conditions was studied. Figure 3 shows the SEM/EDS analyses of the AgNPs-Muc synthesized under the experimental conditions of method 1, at pH 1.5. AgNPs-Muc dominated the precipitate after centrifugation of the reaction mixture at 12,000 rpm. The SEM imagery (Figure 3A) clearly shows the oval shapes of the resulting biogenic AgNPs-Muc, with sizes ranging between 20 and 80 nm. The EDS spectrum provides further evidence of this, with a pronounced, intense peak at 3.00 keV and two lower-intensity peaks at 3.16 and 3.5 keV (Figure 3B). The strong signal at 3.00 keV revealed the presence of metallic silver in the NPs biosynthesized using the protein fraction from *C. aspersum* mucus with MW > 20 kDa in the presence of AsA as an additional reducing agent. The EDS spectrum in Figure 3B shows that the NPs are mainly composed of silver (Ag, 92.85%), oxygen (O, 0.28%), nitrogen (N, 0.95%), carbon (C, 2.28%), and magnesium (Mg, 3.65%).

To determine the size distribution of the nanoparticles, a histogram was constructed by processing a series of SEM images, including data for more than 1000 particles. The results showed that the AgNPs-Muc obtained by method 1 had an almost uniform size distribution around 50 nm.

**Figure 4:**
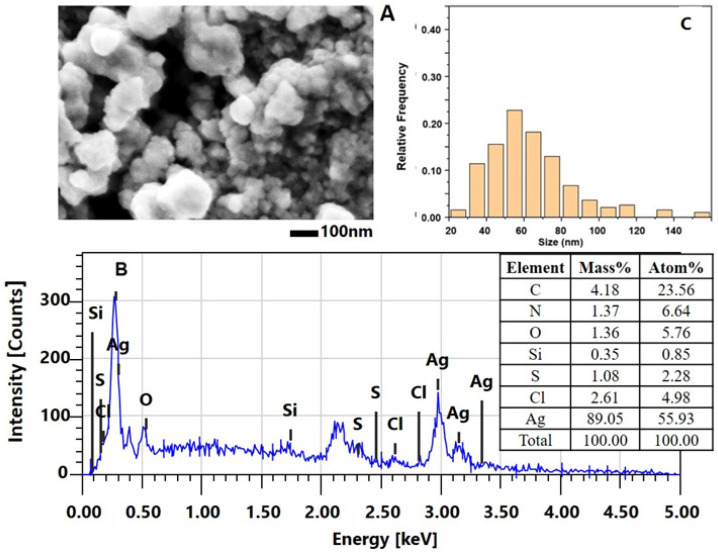
Characterization of AgNPs-Muc obtained using method 2 at pH 3.5. (**A**) SEM image of AgNPs-Muc; (**B**) EDS spectrum of AgNPs-Muc and summary results table; (**C**) Histogram of the particle-size distribution.

Similar results were observed for the nanoparticles synthesized under the experimental conditions of method 2 at pH 3.5 (Figure 4). Titration of the reaction mixture containing the Ag^+^ precursor (AgNO_3_) and the *C. aspersum* mucus with MW > 20 kDa with AsA until reaching pH 3.5 led to well-formed AgNPs-Muc. Figure 4A clearly distinguishes the obtained biogenic AgNPs-Muc, with sizes between 20 and 80 nm and polydispersity, from those obtained using method 1 at pH 1.5.

The histogram of the size distribution of the nanoparticles obtained using method 2 is slightly broader (up to 100 nm) than that of AgNPs-Muc obtained using method 1, although the highest frequency is again around 50 nm (Figure 4B).

In addition, the EDS analysis showed lower-intensity peaks between 3.0 and 3.6 keV (Figure 4C) and a lower concentration of Ag (89.05%) compared to the biogenic AgNPs-Muc obtained using method 1. The EDS spectrum showed the presence of other elements such as oxygen (O, 1.36%), nitrogen (N, 1.37%), carbon (C, 4.18%), sulfur (S, 1.61%), and chloride (Cl, 2.61%).

**Figure 5:**
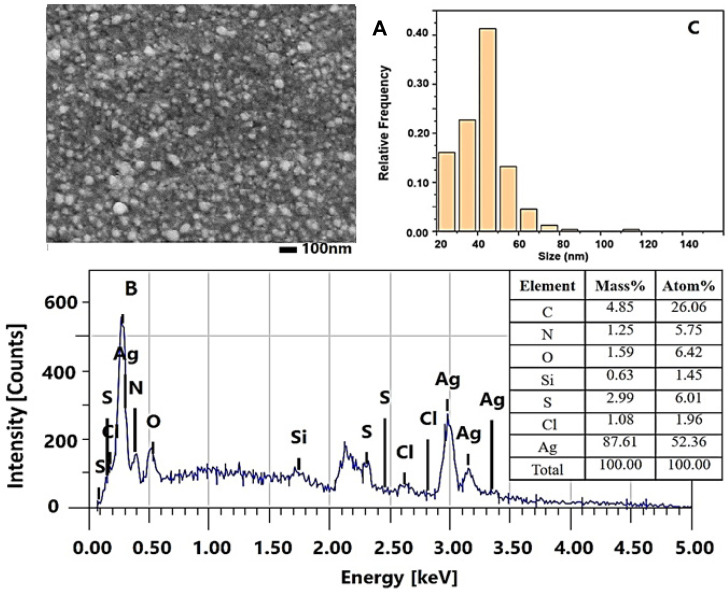
Characterization of AgNPs-Muc obtained using method 3 at pH 7.0. (**A**) SEM image of AgNPs-Muc; (**B**) EDS spectrum of AgNPs-Muc; (**C**) Histogram of the particle-size distribution and summary results table.

Significantly different morphological characteristics were observed for the AgNPs-Muc obtained according to the experimental conditions of method 3 at pH 7.0, as shown in Figure 5. The EDS spectrum of these AgNPs-Muc (Figure 5A) shows smaller-sized nanoparticles compared to those obtained according to methods 1 and 2. On the other hand, the EDS analysis is distinguished by lower peak intensities in the range between 3.0 and 3.6 keV (Figure 5B) and a lower Ag concentration (87.61%) compared to the biogenic NPs obtained at pH 1.5 and pH 3.5.

Low concentrations of other elements, such as oxygen (1.59%), nitrogen (1.25%), sulfur (2.99%), carbon (4.85%), and chloride (1.08%) were also observed. The histogram of the size distribution of the spherical AgNPs-Muc shows a well-defined maximum frequency at 40 nm. A significant characteristic of obtaned AgNPs-Muc is the narrow distribution of a significant amount of particles smaller than 40 nm as well as the negligible amounts of particles larger than 50 nm (Figure 5C).

#### 2.2.3. Characterization of AgNPs-Muc Using Transmission Electron Microscopy (TEM)

**Figure 6:**
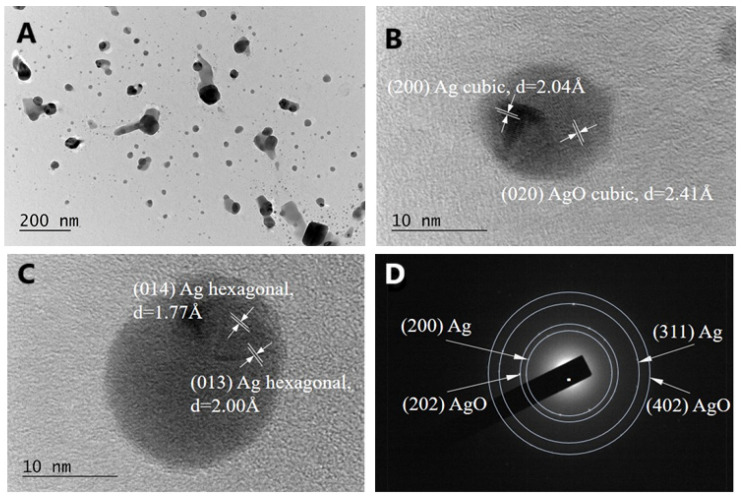
(**A**) Transmission electron microscopy (TEM) image of biogenic Ag and AgONPs synthesized from the mucus fraction of *C. aspersum* with MW > 20 kDa and AsA at pH 1.5; (**B**,**C**) High-resolution TEM images of individual AgNPs; (**D**) Corresponding selected area electron diffraction (SAED) pattern.

The morphology and size of the biogenic AgNPs-Muc synthesized according to methods 1 and 3 were analyzed using TEM. Figure 6 shows the results of the TEM analysis of AgNPs-Muc obtained in the presence of two reducing agents (i.e., the fraction of *C. aspersum* with MW > 20 kDa and AsA) at pH 1.5 (method 1).

The TEM micrograph in Figure 6A clearly reveals the presence of well-defined spherical and quasi-spherical nanoparticles, and these particles are separated and not aggregated. Most of the synthesized AgNPs have a regular spherical shape with a size 20–80 nm, which is in good agreement with the particle sizes calculated from the SEM images.

**Figure 7:**
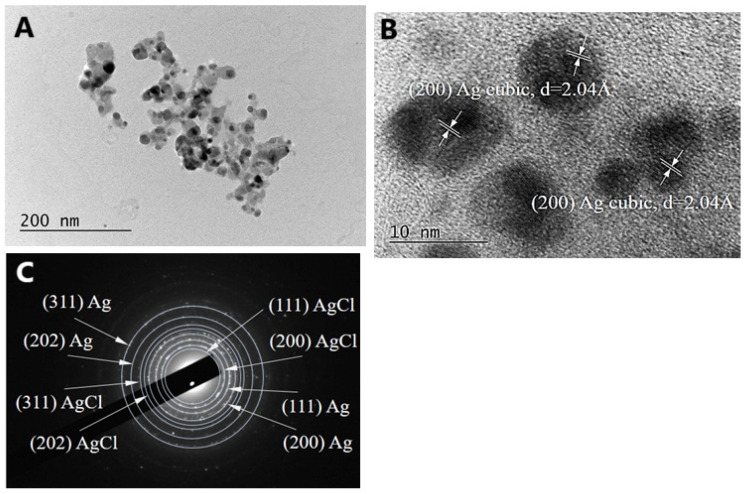
(**A**) TEM image of biogenic Ag and AgONPs synthesized from the mucus fraction of *C. aspersum* with MW > 20 kDa and AsA at pH 7.0; (**B**) High-resolution TEM image of individual AgNPs; (**C**) Corresponding SAED pattern.

High-resolution TEM (HRTEM) analysis was used to determine the structure of the biogenic Ag nanoparticles. Figure 6B,C shows the crystalline structure of single biogenic nanoparticles, with visible lattice fringes. Two forms of Ag were observed—AgO cubic (Figure 6B) and Ag hexagonal (Figure 6C). Additionally, Figure 6D shows a selected area electron diffraction (SAED) pattern, which indicates a polycrystalline nature, with each of the diffraction rings indexed to (111), (202), (402), and (311), corresponding to the fcc crystal structure of metallic silver (JCPDS, No. 04-0783), and a diffraction ring (200) corresponding to the cubic structure of AgK3 (PDF 50-1435). The TEM image of the AgNPs-Muc synthesized in the presence of one reducing agent (i.e., the fraction of *C. aspersum* mucus with MW > 20 kDa) at pH 7.0 is shown in Figure 7. The TEM image clearly reveals the presence of spherical and smaller nanoparticles, with a size range of 20–60 nm, which is in good agreement with the particle sizes calculated from the SEM images.

The HRTEM analyses (Figure 7B) reveals the crystalline structure of a single biogenic nanoparticle and the cubic form of Ag. Additionally, the SAED pattern in Figure 7C shows diffraction rings indexed to (111), (202), and (311), corresponding to the fcc crystal structure of metallic silver (JCPDS, No. 04-0783) and AgCl, as well a diffraction ring (200) corresponding to the cubic structure of AgK3 (PDF 50-1435).

#### 2.2.4. Characterization of AgNPs-Muc Using X-Ray Diffraction (XRD)

**Figure 8:**
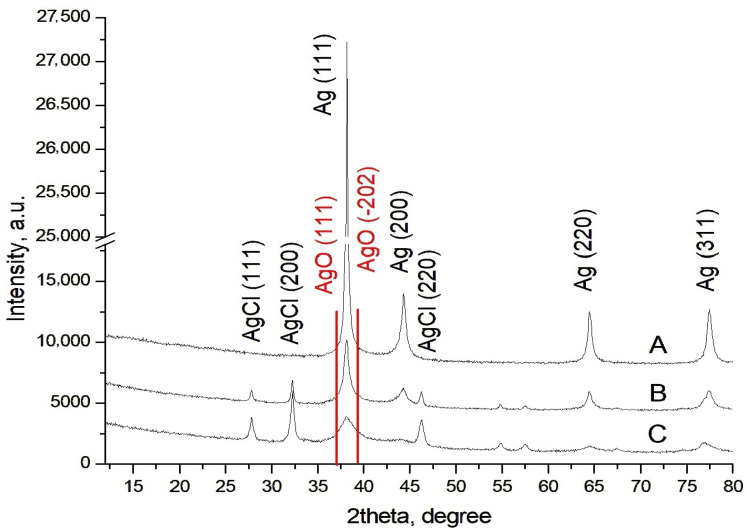
Characterization of AgNPs-Muc using XRD. (**A**) pH 1.5, (**B**) pH 3.5, and (**C**) pH 7.0.

For the detection and characterization of the AgNPs-Muc in the matrix of the mucus fraction with MW > 20 kDa obtained at different pH values (pH 1.5, 3.5, and 7.0), another method was used—XRD analysis. Figure 8 shows the XRD analysis that determined the thickness, phase identification, and crystalline nature of the AgNPs.

Four characteristic diffraction peaks are observed in the spectra of all three methods, at [2θ] = 38.0° (111), 44.27° (200), 64.5° (220), and 76.6° (311), which are most intense in the AgNPs-Muc obtained using method 1 (Figure 8A). These can be indexed as the cubic phase of Ag [JCPDS No. 04-0783]. Along with the diffraction peaks of Ag, secondary diffraction peaks at 2θ = 27.79°, 32.20°, 38.08°, and 46.59° are also observed in the XRD spectra of the AgNPs-Muc obtained using methods 2 and 3 (Figure 3C and Figure 4C). These four peaks indicate the presence of AgO and AgCl in the samples.

#### 2.2.5. Characterization of AgNPs-Muc by Fourier Transform Infrared Spectroscopy (FT-IR)

**Figure 9:**
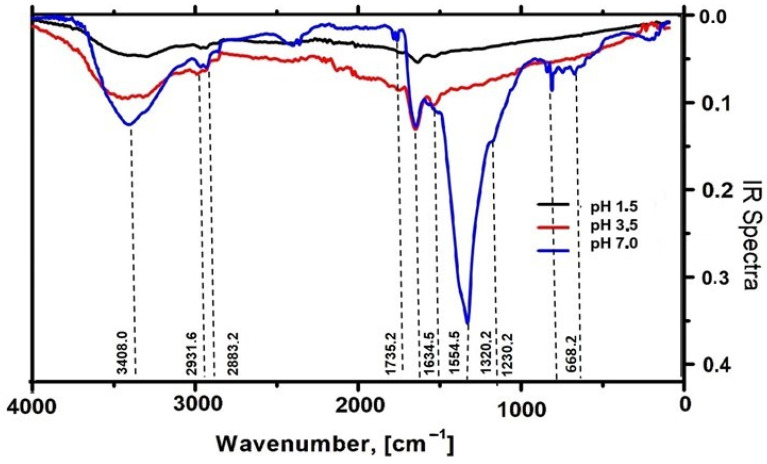
FT-IR spectra of three AgNPs-Muc samples obtained in the matrix of a protein fraction with MW > 20 kDa, as precipitates, at pH 1.5 in the presence of AsA (black line), titrated with AsA to pH 3.5 (red line), and at pH 7.0 in the absence of AsA (blue line).

FT-IR spectral analysis of the AgNPs-Muc obtained as a precipitate using the three different green synthesis methods (as described in Section 4) was carried out in the range 0–4000 cm^−1^. Figure 9 presents a comparative analysis of the FT-IR spectra of the AgNPs obtained from the snail mucus at pH 1.5, pH 3.0, and pH 7.0, indicating different peaks and different behaviors.

The FT-IR spectra of the AgNPs-Muc obtained at pH 7.0, pH 3.5, and 1.5 show absorption peaks in the range 2700–3700 cm^−1^. The peak at 3408.0 cm^−1^, indicated in the three samples, confirms the presence of hydroxyl and phenols. The presence of CH_2_ is reflected by a peak at around 2983 cm^−1^. A peak at 1634.5 cm^−1^ representing the C–C stretching vibrations of alkenes is also observed. The presence of protein macromolecules is expressed by the peaks at 1634.5 cm^−1^ and 1554.5 cm^−1^, assigned to amide I and amide II, respectively. Some peaks are only present in the FT-IR spectra of the AgNPs-Muc synthesized at pH 7.0. For example, the bands at 1735.2 cm^−1^, 1320.2 cm^−1^, and 1230.2 cm^−1^ reveal the stretching vibrations of different groups.

### 2.3. Antibacterial Effect of AgNPs-Muc

**Figure 10:**
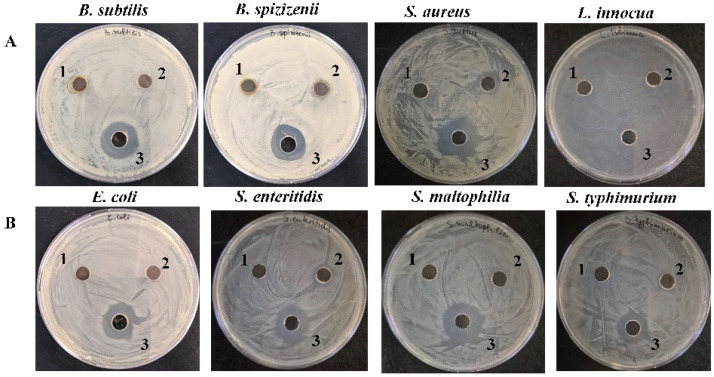
Antimicrobial activity of AgNPs synthesized with a *C. aspersum* mucus fraction with MW > 20 kDa at the reducing agent at different pH values against (**A**) Gram^+^ test microorganisms and (**B**) Gram^−^ test microorganisms.

Initially, the antibacterial effect of AgNPs-Muc obtained at different pH were examined against selected Gram^+^ and Gram^−^ bacterial strains using the well diffusion method. The results showed that the AgNPs synthesized with the *C. aspersum* mucus fraction with MW > 20 kDa at pH 7.0 exhibit a significant broad-spectrum antibacterial effect against all the tested strains, with inhibition zones varying from 17 to 23 mm (Figure 10, Table 1).

In comparison, an antibacterial effect was not observed in for the biogenic metal AgNPs-Muc synthesized at pH 1.5 and 3.0, with the sole exception of a weak inhibitory effect against the Gram^+^ strain *S. aureus* for the AgNPs-Muc synthesized at pH 3.0. The detected antibacterial activity was due mainly to the formed silver nanoparticles, since pure mucus does not show a well-pronounced antibacterial effect, as shown in our previous study [29].

### 2.4. Evaluation of the MIC and MBC of AgNPs-Muc

**Figure 11:**
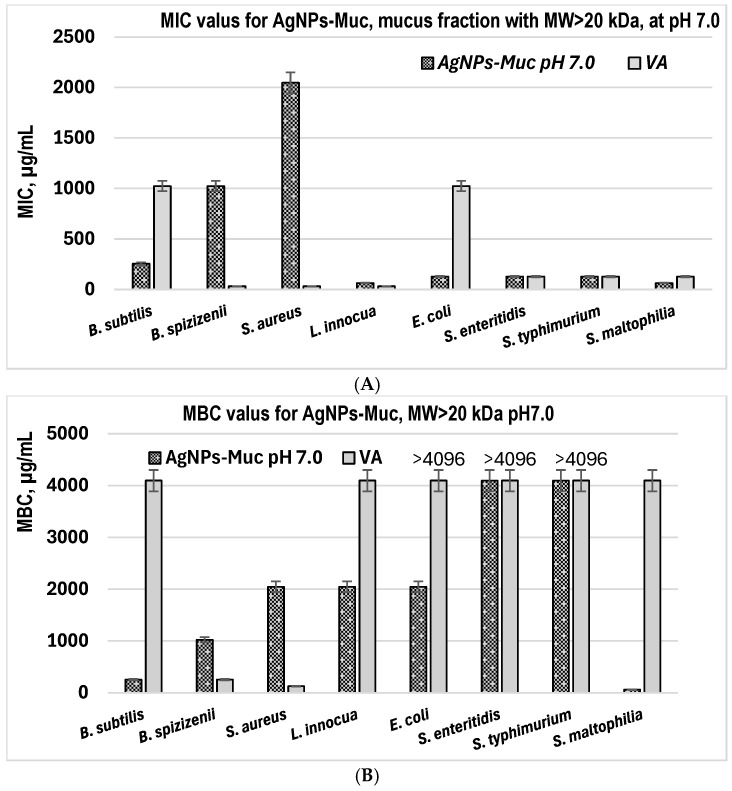
MIC (**A**) and MBC (**B**) values of AgNPs-Muc obtained using the mucus fraction of *C. aspersum* with MW > 20 kDa at pH 7.0 (*p* < 0.05).

The MIC and MBC of the AgNP-Muc obtained at pH 7.0 revealed that the most pronounced antibacterial effect was against *L. innocua* and *S. maltophilia*, where the MIC was 64 μg/mL for both species (Figure 11A).

A significant inhibitory effect against microbial growth was also determined for *E. coli*, *S. typhimurium*, and *S. enteritidis*, with the AgNPs-Muc having a MIC of 128 μg/mL. For the other three strains, the defined MICs varied between 256 and 2048 μg/mL. Nanoparticles formed after reduction with the mucus fraction with MW > 20 kDa at pH 7.0 had a similar effect to the antibiotic vancomycin, and was significantly better in the cases of *B. subtilis* and *E. coli*, for which the MIC for vancomycin is much higher (1024 μg/mL) than that of the AgNPs-Muc (256 and 126 μg/mL, respectively). The detected MBC values were several times higher than the determined MICs, whereas for three strains (*E. coli*, *S. enteridis* and *S. typhimurium*), no such values were acquired (Figure 11B). Comparing the bactericidal effect of vancomycin on cellular growth, this antibiotic exhibited such an effect mainly on the Gram^+^ bacterial strains and only on *S. maltophilia* from the tested Gram^−^ strains. Furthermore, a one-way ANOVA showed a significant difference between the MBC of the AgNP-Muc and VA (F = 5.55, *p* = 0.0335) and no significant difference in their MIC values (F = 0.309, *p* = 0.587). At the same time, the nanoparticles synthesized at pH 7.0, although at higher concentrations, showed broad-spectrum bactericidal activity against most of the bacterial species tested.

The MIC and MBC against *S. aureus* of the AgNPs obtained at pH 3.5 were also determined (Table 2). The data show the significantly weak effect of the AgNPs-Muc, with inhibitory and bactericidal doses that were 32-to-128-times higher than those of VA.

## 3. Discussion

In recent years, green synthesis has emerged as an environmentally sustainable method for producing metal nanoparticles, using biological materials (such as plants, bacteria, and fungi or their extracts) instead of toxic chemicals [34,35,36,37]. The indiscriminate and uncontrolled use of plants in the synthesis of NPs could lead to the extinction of many plant species in nature. Therefore, the use of animal extracts such as the mucus of terrestrial snails is much more advantageous and also includes a number of active compounds [38,39,40]. Various methods have been published for the synthesis of gold [6], silver [22], and copper [29,30] NPs using snail mucus as a reducing agent, which show strong antibacterial activity.

In this paper, we presented the formation of biogenic silver nanoparticles from the active components of the mucus of *C. aspersum*, which enhanced their inhibitory effects on the growth of some bacterial strains compared to the starting fraction.

Since the protein content of the mucus depends on the collection season and the method of mucus extraction, the composition of the *C. aspersum* mucus with MW > 20 kDa as used in this experiment was determined electrophoretically using SDS-PAGE analysis (Figure 1). The protein content with MWs around 26, 30, 35, 37, 45, 56, 80, and 145 kDa was confirmed based on published data from the electrophoretic profiling and analysis with ImageQuantTM TL v8.2.0 software and MALDI [26,29] as well as information for the mucus of the freshwater spiny eel *Mastacembelus armatus* (34, 45, and 144 kDa) [41] and *Achatina fulica* (14.3, 20.1, 29.0, 43.0, 66.0, and 97.4 kDa) [19,22]. Also, electrophoretic analysis showed several bands with MW above 100 kDa, which probably reflect glycoproteins described in the mucus of the snails *Helix aspersa* and *Helix pomatia* [42]. The presence of aromatic residues in the mucus proteins of *C. aspersum* is indicated by the maximum at 280 nm in the UV spectra. These have an important role in the reduction of silver ions, as demonstrated for tyrosine by Xie et al. [43].

The role of peptides and proteins [44,45], ascorbic acid, or sodium borohydride in the reduction of silver ions [29,46,47] has been reported in the literature. Based on this information, AgNPs-Muc were synthesized under different conditions, i.e., medium pH (pH 1.5, 3.5, and 7.0), reducing agents (the *C. aspersum* mucus fraction with MW > 20 kDa and ascorbic acid), and synthesis duration. Determination of the most suitable method for AgNPs-Muc synthesis was achieved after comparing the yield, specific sizes, and properties of the NPs. Tracking the time course of AgNPs-Muc synthesis after 24 h, 48 h, and 72 h demonstrated a maximum at between 420 and 430 nm in the UV–Vis spectra, and the highest yield of AgNPs-Muc was measured after 72 h. Another important factor is the size and shape of the nanoparticles [32,48], as it has been found that AgNPs with smaller sizes have greater antimicrobial efficacy [24,49].

The comparative analysis of the SEM and EDX spectra of the nanoparticles obtained using the three methods showed significant differences in their size and distribution. The best-formed round AgNPs-Muc, with a size range of 20 to 80 nm, were observed at pH 1.5 (Figure 3A), while at pH 3.5, there were also impurities (Figure 4A). A significant difference was observed for the synthesized AgNPs-Muc at pH 7.0, which were less numerous and smaller, ranging between 20 and 60 nm. The average crystal size for the AgNPs-Muc obtained in the presence of AsA, determined using the Debye–Scherer formula, was 50 nm, while in the absence of AsA, it was 40 nm. The influence of the pH of the medium also affected the purity of the obtained product, as the synthesis at a pH of 1.5 produced mostly pure AgNPs, while chloride impurities were observed at pH 3.5 and 7.0.

The characteristics of the NPs were confirmed using TEM, which has two advantages over SEM—better spatial resolution and the possibility of additional analytical measurements [50,51]. Comparative analysis using TEM showed a significant difference in the morphology and size of the biogenic AgNPs; Figure 7A confirms the synthesis of smaller spherical and quasi-spherical AgNPs-Muc in the presence of the *C. aspersum* fraction with MW > 20 kDa at pH 7.0 compared to the obtained NPs at pH 1.5 (Figure 6A).

Another important difference in the structure of AgNPs-Muc was identified by the HRTEM analysis, which confirmed the formation of AgNPs and AgONPs at pH 1.5, while at pH 3.5 and 7.0, the AgNPs contained AgCl impurities, which was confirmed by the X-ray diffraction (XRD) analysis.

Furthermore, the polycrystalline nature of the AgNPs was demonstrated by selected area electron diffraction (SAED), where each of the diffraction rings was indexed to (111), (200), (220), and (311), corresponding to the fcc crystal structure of metallic silver and the cubic structure of AgK3 (PDF 50-1435).

Evidence for the dependence of the degree of crystallinity and particle size on the pH of the medium [52] id given by the XRD diffraction patterns presented in Figure 8, which show a strong dependence of the phase composition on the pH of the synthesis. All samples contained silver nanoparticles, and the best identification match was card number PDF# 01-087-0597 (JCPDS-International Centre for Diffraction Data). In the most acid environment, pure AgNP products were obtained, with no impurity phases. When the pH rose to 3.5 and 7.0, the second phase of silver chloride appeared, identified as PDF#01-71-5290.

The size of the crystallites decreased as the pH of the solution was increased, at 18 nm for sample A, 9 nm for B, and 3–4 nm for C. Typically, crystal size is important for many processes, and the activity of nanoparticles increases as crystal size decreases.

The TEM observations showed the presence of a certain amount of Ag_2_O, although its occurrence was local and the particle number was low. The presence of silver oxide is not ruled out by data obtained using XRD. In Figure 8, the positions of the strongest peaks in AgO PDF #00-043-1038 are shown. The interplanar distances coincide with those calculated from the TEM, with AgO (111) having d = 2.416 and (−202) d = 228.6. It can clearly be seen that these peaks correspond to the main peak for silver. Typically, if AgO particles are low in number and small in size, they will not be visible to conventional XRD.

Only the size of the metal core of the NPs could be estimated using TEM, whereas the combination of the Ag core with the hydrated protein layer in the colloidal solution was determined by FTIR [44,53]. The snail mucus surrounding the core has a dynamic nature, and the groups present in the outer layer of the Ag NP were determined using FTIR. The different behaviors of the AgNPs obtained from the mucus at different pH values are reflected by the FTIR spectra, which show differences in their peaks and intensities. Thus, the FT-IR spectra of the obtained AgNPs at pH 7.0 reflect more peaks with higher intensities compared to those for pH 3.5 and 1.5. The identified peaks correspond to different interactions, with the bands with higher wavenumbers in the range 2700–3700 cm^−1^ (Figure 9) reflecting the presence of CH_2_ around 2883.2 cm^−1^, and 2931.2 cm^−1^ probably from a lipid component in the mucus. The peak at 3408.0 cm^−1^, indicated in the three samples, confirmed the presence of hydroxyl and phenols. The signal reflecting the bound OH- was detected at 3408.0 cm^−1^, and this feature can be attributed to the presence of carbohydrate side chains rather than the protein core. The low-intensity bands observed in the range 2800–3000 cm^−1^ are most likely related to an aromatic core. The benzene moieties appearing at low wavenumbers (1800 cm^−1^ to 2000 cm^−1^) are not well expressed.

The band at 1735.2 cm^−1^, observed only for AgNPs-Muc obtained at pH 7.0, reveals the carbonyl stretching vibrations resulting from the formation of a phenoxide structure due to the oxidation of the phenolic group of tyrosine according [37]. For all samples, but with higher intensity at pH 7.0, a band at 1634.5 cm^−1^ was identified, representing the C–C stretching vibrations of alkenes. In addition, peaks at 1554.5 cm^−1^ and 1320.2 cm^−1^ for the AgNPs-Muc synthesized at pH 7.0 revealed the presence of the CO–C stretching vibrations of carboxyl [6]. The two pronounced absorption bands in the IR spectra at 1634.5 cm^−1^ and 1554.5 cm^−1^ undoubtedly indicate the presence of protein macromolecules, which are attributed to amide I and amide II, respectively [54]. The absorption associated with amide I originates from the stretching vibrations of the C=O bond of the amide [46], while the absorption associated with amide II is mainly due to the bending vibrations of the NH bond. In addition, the band at 1230.2 cm^−1^ is also diagnostic of the presence of amide III proteins, as it originates from C–N stretching [55]. Ester bonds, characteristic of some sugars, are expressed as bands at 1735.2 cm^−1^ and 1230.2 cm^−1^. Similar results were reported by Mane et al. for AgNPs obtained in the matrix of *A. fulica* mucus [22]. These peaks were observed only for the NPs formed at pH 7.0, which are smaller in size. Sugars are likely exposed on the surfaces of the nanoparticles and are responsible for their properties. The second reducer, ascorbic acid, covered the NPs obtained at pH 1.5 and the sugars on their surfaces. The peak located at 668.2 cm^−1^ represents the key component responsible for the reduction and capping of the extract with the metal through their intermolecular interaction [56].

The involvement of the *C. aspersum* mucus fraction with MW > 20 kDa and ascorbic acid as an additional reducing agent in the synthesis of AgNPs differs from its involvement in the synthesis of CuNPs [29,30]. They appear to be a stronger reducer of silver ions, with smaller and more abundant well-formed AgNPs observed.

It is obvious that the three types of AgNPs-Muc synthesized at different pH values significantly differed in their structural properties, especially in size, varying from 50–80 nm (at pH 1.5 and 3.0) to less than 20 nm (at pH 7.0). Ascorbic acid coated and encapsulated nanoparticles. Under acidic pH conditions, AgNPs aggregate and form large nanocomposites; under alkaline pH, the high concentrations of hydroxyl groups on the surfaces of nanoparticles result in repulsive forces in colloidal solutions, preventing their aggregation and reducing their size [57]. On the other hand, at alkaline pH, many functional groups are able to bind silver ions, which makes it possible to obtain smaller AgNPs.

The shape and size of AgNPs are significantly influenced by the experimental parameters used in their synthesis, including temperature, the pH of the solution, the concentration of the Ag(I) compound, and, in the case of biological synthesis, the reducing agent [58]. The most notable property of AgNPs is their shape, which also significantly influences their other characteristics, including their antimicrobial properties. Current knowledge on the mechanisms of AgNPs is limited but it is considered that the impact of AgNPs on cells is based on a number of mechanisms. These include adherence to the bacterial cell wall and membrane, penetration into the cell and disruption of intracellular macromolecules, oxidative stress induction, and signal transduction pathway regulation [59]. AgNP adherence and accumulation on cell surfaces are believed to be facilitated by porins in the outer membranes of Gram^−^ bacteria, which are involved in the passive transport of hydrophilic molecules [60]. As the AgNPs synthesized using the mucus fraction with MW > 20 kD at pH 7.0 were smaller, this supposedly enables their penetration through the bacterial cell wall and membrane, allowing them to exert their antibacterial activity in the cellular cytoplasm. These results are in agreement with other studies that show that the only nanoparticles to exhibit a direct interaction with bacterial cells have a diameter of roughly 1–10 nm [4,61].

Interestingly, many studies on the antibacterial effect of AgNPs state that they are mainly active against Gram^−^ bacterial strains. However, the AgNPs synthesized using the protein fraction of *C. aspersum* mucus with MW > 20 kDa at pH 7.0 showed broad-spectrum antibacterial activity, being significant and effective not only against Gram^−^ strains but also all the tested Gram^+^ strains. Moreover, the efficacy of the obtained biogenic silver nanoparticles was comparable to that of the glycopeptide antibiotic vancomycin, and even better in some cases, like against *B. subtilis* and *E. coli*, with most of the MIC values varying between 64 and 256 μg/mL. Similar results were obtained by Parvekar et al. [62], who estimated that the MIC and MBC of small-sized silver nanoparticles (below 10 nm) against *S. aureus* was 625 µg/mL. Other authors have also confirmed the efficient antibacterial activity against methicillin-resistant *Staphylococcus aureus* of AgNPs with a size range of 10–20 nm obtained by green synthesis and having MIC values ranging between 8.27 and 230 µg/mL. Furthermore, this study showed that at a concentration of 2.046 mg/mL AgNPs-Muc at pH 7.0 also had a bactericidal effect on both Gram^+^ and Gram^−^ bacterial strains. The only antibacterial activity observed for the AgNPs-Muc synthesized at pH 1.5 and 3.0 was against *S. aureus* for those at pH 3.0. This may be due to the high abundance of wall teichoic acids on the surface of *S. aureus*, which allows some of the larger nanoparticles to bind [63]. As a result, the charge in the cell wall is probably disrupted and structural changes occur, leading to limited cell death.

## 4. Materials and Methods

### 4.1. Preparation of Mucus Samples

The native mucus extract was obtained from certified environmentally clean Bulgarian snail farms using a patented technology (BG Utility Model Application Number 2656/08.11.2013) [31]. The protein fraction with MW above 20 kDa, which was used for the synthesis of the AgNPs-Mucs, was obtained by pressure ultrafiltration of purified total mucus extract on a polyethersulfone membrane with a 20 kDa pore size (Microdyn Nadir™ from STER-LITECH Corporation, Goleta, CA, USA, respectively) using an Amicon^®^ Stirred Cell 200 mL (UFSC20001, MerckMillipore, Merck Group, Darmstadt, Germany) connected to an external source of N_2_ [26,32]. The protein concentration was determined using the Bradford method [33].

### 4.2. 1D-Polyacrylamide Gel Electrophoresis (1D-PAGE)

Protein mucus fractions were analyzed using 12% sodium dodecyl sulfate-polyacrylamide gel electrophoresis (SDS-PAGE) according to the Laemmli method with modifications [64] and stained with Coomassie Brilliant Blue G-250. Molecular markers of standard proteins with molecular weights ranging from 6.5 to 200 kDa were used (SigmaMarker^TM^, Sigma-Aldrich, Saint Louis, MO, USA). A commercial Laemmli buffer was used (Leammli Sample Buffer 2x, for SDS PAGE, SERVA Electrophoresis GmbH, Heidelberg, Germany). An equal volume of the sample was mixed with sample buffer, then boiled at 100 °C for 5 min to denature the proteins. Equal volumes containing the samples were dissolved in Laemmli sample buffer, and 20 μg of each sample and protein standard mixture were loaded onto the gel. The process was run at 145 V using a Bio-Rad power supply (Bio-Rad, Hercules, CA, USA).

### 4.3. Synthesis of AgNPs-Muc

Using green synthesis, biogenic AgNPs-Muc were obtained in the mucus fraction matrix with MW > 20 kD at room temperature and at three different pH values of the reaction mixture (the pH was reached by titration with ascorbic acid), as follows:

(1) To the obtained solution of 0.5 g of lyophilized mucus fraction with MW > 20 kDa in 100 mL of distilled H_2_O, 50 mL of a solution of 50 mM AgNO_3_ was added and homogenized for about 40 min on a magnetic stirrer. The obtained solution was titrated to pH 1.5 with a solution of 250 mM ascorbic acid (AsA).
(2) The obtained solution of mucus fraction with MW > 20 kD and 50 mL of a solution of 50 mM AgNO_3_ was titrated to pH 3.5 with a solution of 250 mM AsA.
(3) The green synthesis of AgNPs-Muc was performed at pH 7.0 (without titration by AsA) under the same conditions mentioned above in a solution of 0.5 g of lyophilized mucus fraction with MW > 20 kDa, 100 mL of distilled H_2_O, and 50 mL of 50 mM AgNO_3_ with moderate homogenization on a magnetic stirrer.

In all three methods, the reaction mixture was exposed to sunlight for 30 min. The process of the formation of biogenic nanoparticles in all three methods was monitored using UV–Vis spectra for 3 days under continuous homogenization on a magnetic stirrer.

After the third day, the resulting solutions were centrifuged at 3000 rpm for 5 min to remove coarse particles, and the resulting supernatant was centrifuged again at 12,000 rpm for 15 min to precipitate the AgNPs-Muc. The precipitates were washed three times with distilled H_2_O for 15 min until a neutral pH was achieved. All experiments were performed three times.

### 4.4. Characterization of Biogenic AgNPs-Muc

The following analytical methods and techniques were used to characterize the physicochemical properties of the synthesized AgNPs-Muc: UV–Vis (Shimadzu™ UVmini-1240 Shimadzu Corporation, Kyoto, Japan), FT-IR (Infrared [FT-IR] spectrometer INVENIO-R, Bruker, Karlsruhe, Germany), scanning electron microscopy combined with energy dispersive spectroscopy (SEM/EDS; JEOL JSM6390, Japan and Oxford Instruments, Abingdon, UK), and X-ray diffraction analysis (XRD; goniometer PW1050, Philips, Almelo, The Netherlands).

#### 4.4.1. Characterization of AgNPs-Muc Using UV–Vis Spectroscopy

The absorption spectra of the mucus fraction with MW > 20 kDa and the obtained AgNPs-Muc with and without the presence of AsA were measured in the range 350–750 nm using a UV–Vis spectrometer (Shimadzu™ UVmini-1240) through symmetrical quartz cuvettes (Quartz Glass High Performance 200 nm–2500 nm, Hellma^®^ absorption cuvettes, Hellma GmbH & Co. KG, Müllheim, Bohum, Germany) with an optical length of 10 mm at room temperature (25 °C). Background correction for all spectra was made by subtracting the absorption spectrum of water under the same conditions.

#### 4.4.2. Characterization of AgNPs-Muc Using SEMT

Samples of the AgNPs-Muc obtained at pH 1.5, 3.5, and 7.0 were coated with a thin gold film to avoid sample charging, and the morphologies of the AgNPs-Muc were analyzed under a scanning electron microscope.

The surface morphologies of the AgNPs-Muc obtained under the different conditions and their chemical compositions were characterized in multiple fields of view using scanning electron microscopy. Several dozen individual SEM images of each sample were obtained and used to construct AgNPs-Muc size-distribution histograms.

#### 4.4.3. Characterization of AgNPs-Muc Using X-Ray Diffraction (XRD)

The phase composition of the obtained nanoparticles was determined using an Empyrean X-ray diffractometer system (PANalytical, Almelo, The Netherlands). The device was equipped with an X-ray tube with a copper anode, operating at a voltage of 40 kV and with a current of 40 mA. The experiment was carried out in the range from 10 to 80° 2θ with a step of 0.03°. 2θ and an exposure time of 3 s. The diffraction patterns were analyzed using the HighScore Plus program, using the ICSD (Inorganic Crystal Structure Database) database.

#### 4.4.4. Analysis of AgNPs-Muc Using Transmission Electron Microscopy (TEM)

The analysis was performed with a TEM JEOL JEM 2100 at 200 kV accelerating voltage [JEOL IT800SHL, Tokyo, Japan] An ethanol suspension was prepared for each powder sample, then sonicated and laid on a standard Cu TEM grid coated with amorphous carbon.

#### 4.4.5. Characterization of AgNPs-Muc Using FT-IR

Information on the functional group binding between the AgNPs-Muc obtained under the different conditions in the snail mucus matrix (fraction with MW > 20 kDa) was obtained using an INVENIO-R FT-IR spectrometer (Bruker) with a resolution of 2 cm^−1^. All FT-IR spectra were recorded and accumulated over 120 scans using a diamond crystal IRIS single reflection ATR plate (PIKE Technologies, Fitchburg, WI, USA).

### 4.5. Antibacterial Activity Assessment

#### 4.5.1. Strains and Growth Conditions

Four Gram^+^ (*B. subtilis* NBIMCC 2353, *B. spizizenii* ATCC 6633, *S. aureus* ATCC 6538, and *L. innocua* NBIMCC8755) and four Gram^–^ (*E. coli* ATCC 8739, *S. enteritidis* NBIMCC 8691, *S. typhimurium* ATCC 14028, and *S. maltophilia* ATCC 17666) bacterial strains were used to evaluate the antibacterial activity of the AgNPs obtained using the mucus fractions from the garden snail *C. aspersum* (MW > 20 kDa) synthesized at three different pH values by titration with ascorbic acid (pH 1.5, 3.0, and 7.0). The media utilized for bacterial growth were Nutrient broth (NB) and Nutrient agar (NA) (HiMedia), prepared according to the manufacturer’s directions.

#### 4.5.2. Agar Well Diffusion Assay

The agar well diffusion method was applied for initial antibacterial activity determination [65]. Plates with NA were inoculated with overnight-grown bacterial cultures with a density of 0.5 McFarland. On each plate, wells were pinched by a sterile borer and 100 μL of each AgNP-containing sample was filled into the formed well at a final concentration of 6.4 mg/mL. After incubation at 37 °C for 24 h, the antibacterial activity of the AgNPs-Muc was evaluated as the diameter based on the growth inhibition zones (d, mm).

#### 4.5.3. Evaluation of MIC and MBC

The minimum inhibitory concentration (MIC) values were determined according to the broth microdilution method following the procedure described by the Clinical Laboratory and Standards guidelines [CLSI]. Specifically, 96-well sterile plates were used, and the wells were filled with 180 μL of the nutrient broth media containing the different variants of the AgNPs-Muc at final concentrations of 0, 8, 16, 32, 64, 128, 256, 512, 1024, 2048, and 4096 μg/mL. Wells were inoculated with 20 μL bacterial suspensions corresponding to 0.5 McFarland. Incubation was performed on a Rotor shaker (220 rpm) at 37 °C for 20 h. The MIC values were determined as the lowest concentration of AgNPs-Muc at which bacterial growth is completely inhibited, and the results were additionally confirmed by cell density measurement (600 nm) using a FLUOstar Omega (BMG Labtech, Ortenberg, Germany) microplate reader.

The minimum bactericidal concentrations (MBCs) were evaluated on nutrient agar plates. After determining MICs, 100 μL from each microtiter well without growth was inoculated on agar plates. Incubation was carried out at 37 °C for 24 h, and the MBC values were defined as the concentrations at which no bacterial colony was detected.

As a positive control, the MIC and MBC of commercially available glycopeptide antibiotic vancomycin (VA) were used.

### 4.6. Statistical Analysis

All data are expressed as means ± standard deviation (SD). Statistical analyses were performed using one-way ANOVA, and significant differences were defined as *p* < 0.05.

## 5. Conclusions

In this study, we presented, for the first time, an environmentally friendly method for synthesizing biogenic AgNPs-Muc from a fraction of the mucus of the garden snail *C. aspersum* with MW > 20 kDa. The analyses conducted using UV–Vis, SEM, TEM, EDS, and FT-IR prove that the formation of AgNPs-Muc is dependent on the pH of the medium, as well-developed, 20–80 nm nanoparticles were synthesized under lower pH (1.5 and 3.5) conditions compared to neutral (pH 7.0) conditions, from which fewer but smaller particles (20–60 nm) were obtained. This explains the higher antibacterial activity of the synthesized NPs at pH 7.0, which were smaller in size. This supports the claim that smaller particles are more toxic than larger ones because they have a larger surface area [66].

The results of the in vitro analysis indicate that synthesized Ag/AgONPs offer a new effective antimicrobial agent against high-risk pathogens, as the AgNPs-Muc synthesized using a reducing agent protein fraction from the mucus of *C. aspersum* with MW > 20 kDa at pH 7.0 show a comparable—and in some cases even better—broad-spectrum antibacterial activity comparable to the glycopeptide antibiotic vancomycin.

The Ag/AgONPs synthesized in the presence of AsA were coated with a negative charge, while those synthesized at pH 7.0 had a positive surface charge. These results confirm the dependence of the toxicity of AgNPs on the coating on the surfaces of the nanoparticles [67]. The advantages of positively charged NPs are that they can remain in the bloodstream for longer compared to negatively charged NPs [68], which is a major route for the delivery of anticancer and other agents.

## Figures and Tables

**Table 1:** Growth inhibition zones of Gram^+^ and Gram^−^ bacterial strains in the presence of AgNPs obtained using the mucus fraction of *C. aspersum* with MW > 20 kDa.

	Growth Inhibition Zone (D, mm)			
		Nanoparticle type		
		AgNPs-Muc pH 7.0	AgNPs-Muc pH 3.0	AgNPs-Muc pH 1.5
Gram^+^ strains	*B. subtilis*	20 ± 0.5	0	0
	*B. spizizenii*	18 ± 1.0	0	0
	*S. aureus*	21 ± 1.0	13 ± 0.50	0
	*L. innocua*	19 ± 1.0	0	0
Gram^−^ strains	*E. coli*	19 ± 0.5	0	0
	*S. typhimurium*	17 ± 0.5	0	0
	*S. enteritidis*	19 ± 1.0	0	0
	*S. maltophilia*	23 ± 1.0	0	0

**Table 2:** MIC and MBC values against *S. aureus* ATCC 6538 for AgNPs-Muc obtained using the protein fraction of *C. aspersum* mucus with MW > 20 kDa at pH 3.0.

	MIC (μg/mL)	MBC (μg/mL)
AgNPs-Muc	4096	4096
VA	32	128

## Data Availability

The original contributions presented in this study are included in the article. Further inquiries can be directed to the corresponding authors.
